# Assessment of the Relationship between Maxillary Posterior Teeth and Maxillary Sinus Using Cone-Beam Computed Tomography

**DOI:** 10.1155/2022/6254656

**Published:** 2022-07-05

**Authors:** Alaa Abdelqader Altaweel, Sami Mohammed Saad Sowairi, Ahmed Mohammed Saaduddin Sapri, Sama Abdulelah Saeedi, Asalah Hamad Alamri, Albtoul Ahmed Alnobi, Maha Fahad ALSharif, Ahmed Mohsen A Altokhi, Hisham Abbas

**Affiliations:** ^1^Oral and Maxillofacial Surgery Department, Faculty of Dental Medicine for Boys, Al-Azhar University, Cairo, Egypt; ^2^King Fahd Hospital of the Armed Forces, Jeddah, Saudi Arabia; ^3^Oral and Maxillofacial Surgery Department, Faculty of Dentistry Mansoura University, Mansoura, Egypt; ^4^Batterjee Medical College, Jeddah, Saudi Arabia; ^5^General Dentist, Jeddah, Saudi Arabia; ^6^Alhanan Al-mutamaiz Medical Center, Jeddah, Saudi Arabia; ^7^My Clinic Medical Center, Jeddah, Saudi Arabia; ^8^Deryaq Care Medical, Jeddah, Saudi Arabia; ^9^Department of Oral and Maxillofacial Radiology, Faculty of Dentistry, Cairo University, Cairo, Egypt; ^10^Department of Oral and Maxillofacial Radiology, Vision medical Colleges, Jeddah, Saudi Arabia

## Abstract

**Introduction:**

Because of the close contact between maxillary sinus and maxillary posterior teeth, procedural errors such as perforation of the sinus may occur during surgical intervention resulting in oroantral communication, which if not corrected, would develop into a fistula. The aim of this study was to evaluate the relationship between maxillary posterior teeth and maxillary sinus floor in a population of the western area of Saudi Arabia, and if age, gender, and size may affect such distance.

**Materials and Methods:**

This retrospective study evaluated 539 cone-beam computed tomography (CBCT) radiographs of patients over 20 years of age. Patients were divided into four groups according to age: group I (20–30 years), group II (31–40 years), group III (41–50 years), and group IV (more than 50 years). From coronal and sagittal images of CBCT, the vertical distance between the posterior maxillary root and the maxillary sinus was measured and classified according to its proximity to the maxillary sinus.

**Results:**

Gender and size did not significantly affect the distance between maxillary posterior root and maxillary sinus. However, there was a significant increase in this distance with increased age. Mesiobuccal root of the second molar was the nearest root to the maxillary sinus (0.8 ± 1.62, *p* < 0.001), while the buccal root of the first premolar was the farthest root (5.39 ± 3.26, *p* < 0.001).

**Conclusion:**

Regarding the population of this study, the buccal roots of the second molars are the closest to the sinus floor. Complications associated with maxillary molar extraction and implantation are greater at a younger age. Because the distance between posterior maxillary teeth and maxillary sinus was mostly type 1 (0–2 mm), clinicians are advised to perform CBCT to get a better understanding of the relationship between maxillary posterior roots and maxillary sinus before surgical intervention.

## 1. Introduction

The maxillary sinus (MS) is a vital anatomic structure located above the maxillary posterior teeth (MPT) and adjacent to the nasal cavity. At the age of 20, the maxillary sinus floor (MSF) is created by the alveolar process of the maxilla and is located about 5 mm below the level of the nasal floor [1, 2]. The volume of the MS cavity is a dynamic process that increases to the same horizontal level as the nasal floor at 12 years of age and then drops slightly below the level of the nasal cavity with the eruption of the upper third molar at 20 years of age [3]. Extraction of the maxillary tooth, particularly many neighboring teeth, can also affect MS pneumatization [1–4].

Because of the close contact between MSF and MPT root apices, odontogenic infection can migrate directly to the MS creating odontogenic maxillary sinusitis, which accounts for 10 to 12 percent of all sinusitis [5, 6]. In addition, procedural errors such as perforation of the sinus may occur during surgical intervention resulting in oroantral communication. Also, inappropriate implant placement may be associated with a pathological alteration of the sinus [7]. All preceding conditions might result in a variety of complications, which can be difficult to be managed [8, 9].

Plain X-rays as periapical and orthopantomogram are conventional imaging methods used for investigating the relationship between MPT and MSF. Because these methods are a two-dimensional projection, it may result in an inaccurate diagnosis [10]. In the last two decades, cone-beam computed tomography (CBCT) has become a common maxillofacial radiological method. CBCT is a cross-sectional imaging technique that is useful for clinical diagnosis and developing successful treatment plans. When compared to multidetector CT, CBCT uses less radiation, has a higher resolution, and takes less time to scan. CBCT can clearly analyze the relation between the maxillary root apices and MS by providing high-quality three-dimensional (3D) pictures of the oral and maxillofacial areas [11].

The relationship between MPT and MSF has been studied in the past. Von Arx et al. investigated the distance between maxillary premolar roots and MSF in Swiss people and reported that gender, age, size, and the presence or absence of premolars have no significant effects on the mean distance between premolar roots and the MSF [12]. Ok et al. measured the distance between MPT root apex and MSF in Turkish people and found no significant differences between size measurements but this distance is significantly affected by the age decade [13]. In the study of Anter et al., the investigators reported that, in Egyptian subjects, the buccal roots of maxillary second molars are highly anticipated for MSF invagination. The lower the age, the closer are the maxillary molar roots to the MSF [14].

However, few studies have investigated the relationship between MPT and MSF in Saudi people, and if gender, age, and size may affect the distance between root apex and MSF is also unclear. Therefore, this study was designed to evaluate the anatomic relation of MPT apex to MSF in a population of western area of Saudi Arabia and if age, gender, and size may affect such a distance.

## 2. Materials and Methods

### 2.1. Study Design

This retrospective study was performed on patients visiting the outpatient dental clinics and government hospitals during the period from February 2020 to February 2021. The study was conducted according to the rules of ethics declared by Helsinki, and ethical committee approval was obtained from the educational institutions on November 2019 (no. 19–11/5). Personal information of the patients was not identified, and only the investigators had access to the records.

### 2.2. Sampling and Sample Size

A sample size calculation was performed using the Raosoft sample size calculator (http://www.raosoft.com/samplesize.html) based on the standard deviation set at 1.96 for 95% confidence interval, 5% margin of error, anticipated response (eligible patients' records fulfilling the inclusion criteria) of 50%, and total population size in the Makkah region, within the age range of the study, of 5,666,004 (according to the General Authority for Statistics: https://www.stats.gov.sa/ar/1007-0). This calculation gave a minimum sample size required for 385 of CBCT scans for Saudi subjects to be included in the study.

### 2.3. Population and Inclusion Criteria

Selection criteria of the scans to be included in the study was based on the following: the scans were for Saudi individuals, their age ranged between 20 and 60 years old and with completely erupted maxillary teeth with fully formed roots free from any apical lesion. While scans were excluded if there were any changes associated with pathologic lesions in the maxillary posterior region or in the maxillary sinus, the presence of signs of the previous surgery in the MS, or the presence of metallic artifacts precluded visibility of the maxillary molars' apices or MSF.

Patients were divided into 4 age groups: group I (20–30 years), group II (31–40 years), group III (41–50 years), and group IV (more than 50 years). This study's data included the following: (1) demographic information such as name, age, and gender. These data were tabulated for each scan, labeled with a number, and kept hidden from radiographic evaluation investigators. –(2) The radiographic data included CBCT images, which were processed by Dental Imaging Software version 6.14.7.3.

### 2.4. Procedures

**Figure 1:**
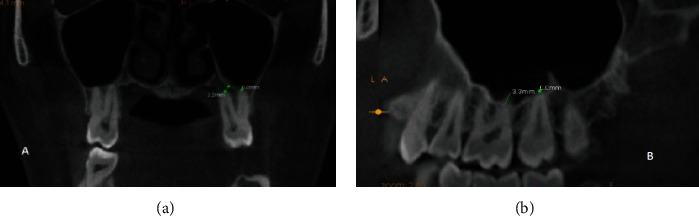
(a) Coronal section showing MB USM 2.2 from MSF and P USM contacting MSF. (b) Sagittal section of DB UFM 3.3 MM from MSF.

Coronal and sagittal CBCT images with 0.4 mm slice thickness and 0.4 mm interslice distance were used for measuring vertical distance in millimeters (mm) between the roots of MPT and MSF, after being corrected to be passed through a long axis of the root under evaluation. This evaluation was performed according to previous studies [11, 15–17] where the shortest vertical distance between the root of each MPT and the border of MSF was measured in serial sagittal and coronal. A negative value was registered if the apex of the root penetrated into the MSF (Figure 1). Only one value was recorded if the roots were fused. The relationship between the distance of MPT and MSF was analyzed regarding patient's age, gender, and size.

To ensure the reliability of values, the measurements obtained from CBCT images were evaluated by primary investigators. Intraobserver variations were expected, so a second-step verification was performed by the same investigators by randomly assessing 20% of the images 2 weeks later (with blind knowledge of the initial measurements) to ensure that there was no significant difference between the mean values of the two measurements taken by the investigators.

The vertical distance between MPT and MSF was classified, as described by Didilescu et al [18]. Distances 0, 0–2, 2–4, 4–6, and >6 mm were classified as type 0 to 4. “Type 0” with excessive sinus pneumatization was considered as a highly risk group, “Type 1” as approximated to the sinus was considered as a risky group, and “Type 2” was considered as a less risky group, while “Type 3” and “Type 4” as no sinus approximation, so it was considered as a nonrisky group.

### 2.5. Statistical Analysis

Statistical analysis was performed using the SPSS computer package (IBM SPSS Statistics for Windows, version 25.0. Armonk, NY : IBM Corp., USA). For descriptive values, the mean ± SD was used for quantitative variables, while frequency and percentage were used for qualitative variables. Mann–Whitney and Kruskal–Wallis tests were used to assess the differences in means of quantitative nonparametric variables. The statistical methods were confirmed, presuming a significant level of *p* < 0.05 and a highly significant level of *p* < 0.001.

## 3. Results

In total, 4000 MPT were evaluated, 400 from each tooth, upper third molar (UTM) evaluated from 539 patients (most prevalent extracted tooth), upper first molar (UFM) evaluated from 488 patients, upper first premolar (UFP) evaluated from 440 patients, upper second premolar (USP) evaluated from 435 patients, and upper second molar (USM) were evaluated from 322 patients (less prevalent extracted tooth).

The mean distance of different roots from MSF showed that MB USM was the nearest root to MSF (*p* < 0.001) followed by the distobuccal root of the upper second molar (DB USM) and then the palatal root of the upper first molar (P UFM), while the farthest root from MSF was the buccal root of the upper first premolar (B UFP) (*p* < 0.001) followed by the palatal root of the upper first premolar (PR UFP) and then the root of the upper second premolar. There were no statistically significant differences between males and females or between the right and left sides regarding the distance between the roots of MPT and MSF (Table 1). However, the mean distance of different roots from MSF showed a significant increase with the increase in age (Table 2).

**Figure 2:**
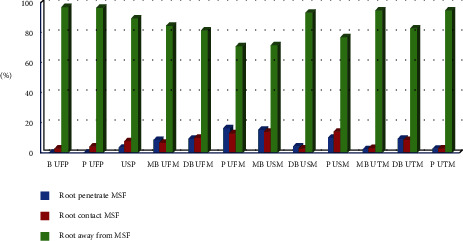
Distribution of different roots in relation to distance from maxillary sinus floor.

The majority of different roots (70%–97%) were distanced away from MSF (mainly B UFP and P UFP), about 2.5%–14% of roots were in contact with the MSF (mainly MB USM and palatal of the upper second molar (P USM), while about 0%–16% were penetrating the MSF, mainly P UFM and MB USM (Figure 2).

**Figure 3:**
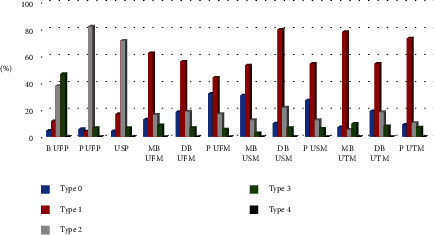
Percent of maxillary posterior roots according to Didilescu classification.

In this study, a root distance from MSF “Type 1” is the most prevalent type (49.1%). Excessive sinus pneumatization “Type 0” was obvious in palatal root of the upper first molar (P UFM) and MB USM and then P USM, and “Type 1” was obvious in DB USM, mesiobuccal of the upper third molar (MB UTM), palatal of the upper third molar (P UTM), and distobuccal of the upper first molar (DB UFM) which are considered the most risky groups. Sinus approximation “Type 2” was obvious in P UFP, and the upper second molar root is considered the less risky group, while no approximation “Type 3” was seen in B UFP which are considered nonrisky groups (Figure 3).

The mean distance between the roots of MPT and MSF was increased in the older age group when compared with the youngest age group. This increased distance was maximally related to P USM by 1.3 (0.27) mm (increased by 142.86%), followed by mesiobuccal upper first molar (MB UFM) by 1.51 (3.96) mm (increased by 117.97%), and the least increase was related to P UTM by 0.35 (2.86) mm (increased by only 21.21%) (Table 3).

## 4. Discussion

Understanding the anatomical relationship between MS and MPT is critical not only for surgical treatments such as tooth extraction, implant implantation, and sinus lifting but also for the perception of pulpal illnesses spreading into the maxillary sinus and orthodontic movement such as tooth intrusion. The maxillary sinus floor may extend between the roots of the maxillary posterior teeth, causing the apices roots to protrude into the sinus. Following tooth extraction, there is a risk of pneumatization, which reduces the amount of bone availability for dental implant installation [17].

Based on the differences in genetic properties of different populations, this study utilized CBCT to assess the distance between MPT and MSF in the population of the western area of Saudi Arabia.

Results of this study demonstrated that MB USM was the nearest one to MSF then DB USM followed by P UFM. While the farthest root from MSF was B UFP, then P UFP, followed by the root of the upper second premolar. These results were in agreement with Junk et al. [11], Poorebrahim et al. [18], and Pei et al. [19], while it was in contrast to Didilescu et al. [20], Shokry et al. [21], and Hameed et al. [22].

Didilescu et al. [20] and Shokry et al. [21] investigated only UFM; so, their results were different from those of the present study. Hameed S et al. studied a population from Al-Qassim population of Saudi Arabia and found DB USM was the closest to the sinus floor, and difference between their findings and our study might be justified by an environmental factor [22].

The present study showed no significant difference between the right and left sides. Similar results were obtained by Kiliey et al. [23], Kilic et al. [16], and Shokry et al. [21]; the present study showed no significant difference between males and females. These results were in accordance with the results obtained by Kilic et al. [16] and Shokry et al [21], while in disagreement with those reported by Shokri et al. [24] who studied a population from Hamedan, Iran, and reported the protrusion of roots into the sinus was more prevalent in males [24]. This difference between the two studies can be justified by the different shapes of the maxilla in males and females of different ethnicities.

Results of the current investigation demonstrated that there was a significant increase in the mean distance of different roots from the MSF with increasing age. This increased a distance between the PMR and the MSF with advancement in age can be explained by the physiologic tooth eruption that compensates the reduction in clinical crown associated with aging. Some investigators have suggested that, after the development of MS, the maxillary sinus volume will be decreased and the MSF will move upwards, unless interference is encountered (e.g., tooth extractions that cause sinus pneumatization) [25–27]. The present study revealed that the distance between molar roots and MSF increased with age, which indicated that surgical complications associated with tooth extraction or implant installation were higher in adolescents.

This result is in agreement with Elsayed et al. [28], Shubhasini et al. [29], and Arji et al. [5]. In contrast to Didilescu et al. [20] and Tafakhori et al. [30], Didilescu et al. [20] only studied UFM, while the study of Tafakhori et al. [30] studied small sample size that consists of 35 CBCT radiographs belonging to patients aged 20 years or older, and different ethnicities of the population can explain the difference between the results.

Based on Didilescu et al.'s [20] classification, the distance between MPT and MSF in the western area of Saudi Arabia was mostly type 1. This is in agreement with results of Shokry et al. [21] and Shubhasini et al. [29]. In contrast to Didilescu et al. [20], who studied only UFM, and Tafakhori et al. [30], different ethnicities of the participants can interpret the difference between the latter author and our study.

### 4.1. Limitations of the Study

This study included only Saudi population in the western area; it is recommended to do the same study on different populations to get more generalized results.

## 5. Conclusion

Regarding the population of this study, the buccal roots of the upper second molar are the closest to the sinus floor. Complications associated with maxillary molar extraction and implantation were greater at a younger age. Because the distance between the roots of posterior maxillary teeth and maxillary sinus floor was mostly type 1 (0–2 mm) in a population of western area of Saudi Arabia; clinicians are advised to perform CBCT to get a better understanding of the relationship between tooth root maxillary sinus before any surgical intervention.

## Figures and Tables

**Table 1:** Mean distance of different maxillary posterior roots from MSF among the studied sample.

Roots	Total number of patients			Male	Female	*P* value	Rt	Lt	*P* value
	Mean in mm (SD)	Min–max	95% CI	Mean in mm (SD)	Mean in mm (SD)		Mean in mm (SD)	Mean ± in mm (SD)	
B UFP	5.39 (3.26)	−0.8–17.9	5.16–5.61	5.56 (3.70)	5.24 (2.80)	0.160	5.49 (4.06)	5.27 (3.11)	0.385
P UFP	5.01 (3.23)	0.0–18.3	4.78–5.24	4.79 (2.70)	5.21 (3.63)	0.069	4.66 (3.14)	5.08 (3.52)	0.075
USP	2.9 (2.81)	−4.5–18.9	2.7–3.09	3.02 (2.98)	2.78 (2.64)	0.222	2.95 (2.11)	3.21 (2.75)	0.134
MB UFM	1.95 (2.2)	−4.2–12.3	1.79–2.11	1.86 (2.04)	2.03 (2.33)	0.278	1.92 (2.03)	2.16 (2.24)	0.113
DB UFM	1.63 (2.21)	−5.4–14.9	1.47–1.78	1.70 (2.29)	1.57 (2.13)	0.389	1.74 (2.17)	1.68 (2.06)	0.688
P UFM	1.36 (2.47)	−6.2–16.9	1.19–1.54	1.32 (2.34)	1.40 (2.58)	0.656	1.44 (2.41)	1.37 (2.56)	0.691
MB USM	0.8 (1.62)	−7.0–12.7	0.69–0.92	0.83 (1.51)	0.78 (1.71)	0.722	0.69 (1.45)	0.88 (1.8)	0.101
DB USM	1.14 (1.6)	−5.1–12.7	1.02–1.25	1.19 (1.65)	1.09 (1.56)	0.410	1.26 (1.88)	1.04 (1.51)	0.068
P USM	1.47 (2.06)	−9.2–13.0	1.32–1.61	1.51 (2.27)	1.43 (1.85)	0.610	1.6 (2.18)	1.48 (1.92)	0.409
MB UTM	1.76 (1.94)	−5.2–12.9	1.62–1.89	1.85 (1.96)	1.67 (1.92)	0.185	1.85 (1.96)	1.67 (1.92)	0.648
DB UTM	1.75 (2.11)	−4.0–13.5	1.61–1.9	1.72 (2.25)	1.79 (1.97)	0.639	1.67 (2.05)	1.81 (2.16)	0.347
P UTM	1.85 (1.91)	−5.8–14.3	1.71–1.98	1.92 (1.89)	1.78 (1.92)	0.325	1.84 (1.77)	1.96 (2.05)	0.376

**Table 2:** The mean distance of different maxillary posterior roots from MSF stratified by age groups.

Roots	Group I	Group II	Group III	Group IV	*P* value
	Mean in mm (SD)	Mean in mm (SD)	Mean in mm (SD)	Mean in mm (SD)	
B UFP	4.77 (2.86)	4.8 (2.29)	5.87 (3.36)	6.11 (4.05)	<.001^*∗*^
P UFP	4.22 (2.24)	4.54 (1.94)	5.42 (3.64)	5.86 (4.26)	<.001^*∗*^
USP	2.36 (2.53)	2.67 (2.81)	3.16 (2.94)	3.4 (2.84)	.001^*∗*^
MB UFM	1.28 (1.42)	1.55 (2.28)	2.19 (1.45)	2.79 (2.95)	<.001^*∗*^
DB UFM	1.26 (1.72)	1.47 (2.58)	1.77 (2.76)	2.02 (1.38)	0.004^*∗*^
P UFM	0.95 (1.49)	1.15 (2.38)	1.51 (2.06)	1.84 (3.45)	0.001^*∗*^
MB USM	0.62 (1.59)	0.67 (2.02)	0.86 (1.24)	1.07 (1.48)	0.021^*∗*^
DB USM	0.98 (0.93)	1.03 (1.39)	1.2 (1.02)	1.34 (2.52)	0.101
P USM	0.91 (1.84)	1.28 (1.18)	1.47 (2.68)	2.21 (2.03)	0<.001^*∗*^
MB UTM	1.41 (0.82)	1.65 (1.71)	1.93 (1.21)	2.03 (3.13)	0.006^*∗*^
DB UTM	1.29 (2.0)	1.5 (1.32)	1.6 (2.65)	2.62 (1.98)	0<.001^*∗*^
P UTM	1.65 (1.52)	1.86 (1.05)	1.91 (1.8)	2.0 (2.8)	0.313

^*∗*^: significant.

**Table 3:** The mean and percentage change (increase) in the distance between different maxillary posterior roots and MSF among age groups.

Roots		Groups I and II	Groups II and III Mean (SD) (%)	Groups III and IV Mean (SD) (%)	Groups I and IV Mean (SD) (%)
B UFP	Mean (SD)	0.03 (3.46)	1.07 (4.02)	0.24 (2.34)	1.34 (3.68)
	Percent (%)	0.63	22.29	4.09	28.09

P UFP	Mean (SD)	0.32 (2.74)	0.88 (3.65)	0.44 (3.71)	1.64 (4.32)
	Percent (%)	7.58	19.38	8.12	38.86

USP	Mean (SD)	0.31 (3.52)	0.49 (3.82)	0.24 (2.94)	1.04 (3.34)
	Percent (%)	13.14	18.35	7.59	44.07

MB UFM	Mean (SD)	0.27 (2.68)	0.64 (2.69)	0.6 (2.06)	1.51 (3.96)
	Percent (%)	21.09	41.29	27.40	117.97

DB UFM	Mean (SD)	0.21 (3.12)	0.3 (3.73)	0.25 (3.25)	0.76 (3.54)
	Percent (%)	16.67	20.41	14.12	60.32

P UFM	Mean (SD)	0.2 (1.49)	0.36 (3.24)	0.33 (2.83)	0.89 (3.01)
	Percent (%)	21.05	31.30	21.85	93.68

MB USM	Mean (SD)	0.05 (2.51)	0.19 (2.43)	0.21 (3.26)	0.45 (2.91)
	Percent (%)	8.06	28.36	24.42	72.58

DB USM	Mean (SD)	0.05 (1.58)	0.17 (1.81)	0.14 (2.04)	0.36 (3.82)
	Percent (%)	5.10	16.50	11.67	36.73

P USM	Mean (SD)	0.37 (2.36)	0.19 (2.96)	0.74 (2.88)	1.3 (3.27)
	Percent (%)	40.66	14.84	50.34	142.86

MB UTM	Mean (SD)	0.24 (1.80)	0.28 (2.17)	0.1 (2.12)	0.62 (3.34)
	Percent (%)	17.02	16.97	5.18	43.97

DB UTM	Mean (SD)	0.21 (2.49)	0.1 (3.06)	1.02 (4.35)	1.33 (4.03)
	Percent (%)	16.28	6.67	63.75	103.10

P UTM	Mean (SD)	0.21 ± 1.90	0.05 ± 2.04	0.09 ± 2.0	0.35 ± 2.86
	Percent (%)	12.73	2.69	4.71	21.21

## Data Availability

The data used to support the findings of this study are available with the corresponding author on reasonable request.

## Abbreviations

MS: Maxillary sinus
MPT: Maxillary posterior teeth
MSF: Maxillary sinus floor
UFP: Upper first premolar
USP: Upper second premolar
UFM: Upper first molar
USM: Upper second molar
UTM: Upper third molar
B UFP: Buccal root of the upper first premolar
PR UFP: Palatal root of the upper first premolar
MB UFM: Mesiobuccal root of the upper first molar
DB UFM: Distobuccal root of the upper first molar
P UFM: Palatal root of the upper first molar
MB USM: Mesiobuccal root of the upper second molar
DB USM: Distobuccal root of the upper second molar
P USM: Palatal root of the upper second molar
MB UTM: Mesiobuccal root of the upper third molar
DB UTM: Distobuccal root of the upper third molar
P UTM: Palatal root of the upper third molar.

## Supplementary Materials

Supplementary Materials This study was performed according to the STROBE checklist (Supplementary file). All figures and tables were included as supplementary files.
